# SARC-HD STUDY: another step forward in implementing a Brazilian multicenter research infrastructure of informative clinical trials on renal replacement therapies?

**DOI:** 10.1590/2175-8239-JBN-2025-E002en

**Published:** 2025-03-21

**Authors:** José Carolino Divino-Filho

**Affiliations:** 1 Karolinska Institutet, CLINTEC, Division of Renal Medicine, Stockholm, Sweden.

Brazil, with an area of 8,500,000 km^2^, has more than 200 million inhabitants, is divided into five large regions with economic, cultural and geographic differences, and is one the ten largest economies in the world.

There is a scarcity of epidemiological data on the situation of kidney disease in the country as well as on the economic, administrative, and management aspects of the line services associated with renal replacement therapy (RRT) as a whole.

Observational studies, which can be exploratory or confirmatory in nature, serve as an important substrate for generating interesting ideas to be tested in appropriate clinical studies in order to generate medical evidence.

Sarcopenia, an age-related condition characterized by a degenerative loss of skeletal muscle mass, quality and strength, is considered a component of frailty syndrome^1^. Moreover, sarcopenia can lead to reduced quality of life, falls, fracture and disability^2,3^.

The new ICD-10-CM (M62.84) code for sarcopenia represents a major step forward in recognizing sarcopenia as a disease^4^, thereby increasing the avail­ability of diagnostic tools and interest in developing multicentre studies. Regarding sarcopenia and patients on hemodialysis (HD), the Brazilian Dialysis Survey (BDS), an annual national survey of Brazilian dialysis centers^5^, showed that only 37% of HD dialysis centers included sarcopenia-related assessments in routine practice. The BDS 2022 data confirmed that the prevalence of HD dialysis patients increased steadily over the years, with a growing number of patients receiving hemodiafiltration. Moreover, the mortality rate decreased to similar levels as the pre-COVID-19 pandemic period^5^.

The SARC-HD protocol has been previously published and its detailed information can be found elsewhere^6^. In this issue of BJN, Duarte et al^7^ describe the experience during the recruitment and implementation phases of a cohort study, SARC-HD, conducted in 19 Brazilian HD centers. After completion of the recruiting (centers and patients) and implementation phases, a structured qualitative and quantitative questionnaire was distributed to collect feedback from the principal investigators. From a total of 1525 HD patients screened, 1008 were enrolled in the SARC-HD multicentre study in order to explore sarcopenia in HD centers nationwide.

The objective of this initial report is to describe the experience with the recruitment of centers and patients as well as with the implementation of the SARC-HD study. According to the authors, most of the invited centers were able to participate in and complete the implementation phase. Their perception was that the development of standardized tutorials and materials were useful facilitators, whereas some centers described the 7p-SGA as difficult to apply, especially when not performed by an experienced nutritionist.

Systematic data collection of patients undergoing renal replacement therapy is critical to the epidemi­ological and clinical understanding of the treatment. These data may allow a more rational use of economic resources and identify interventions to improve treatment and decrease morbidity and mortality of these patients.

The Brazilian Peritoneal Dialysis Multicenter Study (BRAZPD), an observational cohort study of peritoneal dialysis (PD) in Brazil that collected monthly demographic, clinical, laboratory, and outcome data of PD patients treated in 114 dialysis clinics in the country, was started in December 2004 and ended in September 2011. BRAZPD has generated solid and important information that has been very useful as a reality check of the PD therapy for all Brazilian PD centers.

The BRAZPD has encouraged young nephrologists and HCP to work with the data and this has led to several papers (some of which are cited here)^8,9,10,11^, in addition to Master’s degrees and PhD thesis in different universities and states of the country.

Bringing together health care professionals (HCP) from different areas in Brazil to participate in more meaningful clinical trials is not a dream. It is time to design and conduct multicenter studies that can directly improve care of patients participating in the trials as well as all patients treated in the same centers.

I fully agree with the authors when they state that “despite limited research resources and funding in countries such as Brazil, multicenter research may be feasible if they are properly designed”.

According to the authors, the main challenge ahead is the lack of standardized information in electronic medical records. I pose them a question: “Have you thought about joining forces with other groups that have conducted or are conducting clinical studies in the area of renal replacement therapies and sharing tools and experiences?”

The experience from the SARC-HD study will provide information and evidence of significant clinical applicability. It is very interesting to point out that in the paper by Duarte et al^7^ all principal investigators considered *publishing scientific articles and learning/exchanging knowledge* as important outputs of their participation in the SARC-HD study. The same attitude/position was expressed by most of the investigators in the BRAZPD study.

Meanwhile, SARC-HD is certainly contributing to the understanding of sarcopenia at the national level and, not least, raises specific sarcopenia questions that may lead to the development of cooperative studies among the different national and regional registries in Brazil and around the world.

Congratulations for the authors!
