# Supplementary material for: SARC-HD STUDY: another step forward in implementing a Brazilian multicenter research infrastructure of informative clinical trials on renal replacement therapies?
Source: J Bras Nefrol. 2025 Mar 21;47(1):e2025E002. doi: 10.1590/2175-8239-JBN-2025-E002en (PMC11931861; doi:10.1590/2175-8239-JBN-2025-E002en)
Supplement: Translation

# Translation: Estudo SARC-HD: mais um passo em direção à implementação de uma infraestrutura de pesquisa multicêntrica brasileira de ensaios clínicos informativos sobre terapias renais substitutivas?

**DOI:** 10.1590/2175-8239-JBN-2025-E002pt

**Authors:** José Carolino Divino-Filho

**Affiliations:** 1 Karolinska Institutet, CLINTEC, Division of Renal Medicine, Stockholm, Sweden.

O Brasil, com uma área de 8.500.000 km^2^ e mais de 200 milhões de habitantes, é dividido em cinco grandes regiões com diferenças econômicas, culturais e geográficas, sendo uma das dez maiores economias do mundo.

Existe uma escassez de dados epi­demiológicos sobre a situação da doença renal no país, bem como sobre os aspectos econômicos, administrativos e de gestão das linhas de serviços associados à terapia renal substitutiva (TRS) como um todo.

Estudos observacionais, que podem ser de natureza exploratória ou confirmatória, servem como um substrato importante para a geração de ideias interessantes a serem testadas em estudos clínicos adequados, a fim de gerar evidências médicas.

A sarcopenia, uma condição rela­cionada à idade caracterizada por uma perda degenerativa da massa, qualidade e força do músculo esquelético, é con­siderada um componente da síndrome da fragilidade^1^. Além disso, a sarcopenia pode levar à redução da qualidade de vida, quedas, fraturas e incapacidade^2,3^.

O novo código CID-10-CM (M62.84) para sarcopenia representa um grande avanço no reconhecimento da sarcopenia como uma doença^4^, aumentando assim a disponibilidade de ferramentas diagnósticas e o interesse no desenvolvimento de estudos multicêntricos. Em relação à sarcopenia e aos pacientes em hemodiálise (HD), o Censo Brasileiro de Diálise (CBD), uma pesquisa nacional anual dos centros de diálise brasileiros^5^, mostrou que apenas 37% dos centros de HD incluíram avaliações relacionadas à sarcopenia na prática de rotina. Os dados do CBD 2022 confirmaram que a prevalência de pacientes dialíticos em HD aumentou de forma constante ao longo dos anos, com um número crescente de pacientes recebendo hemodiafiltração. Além disso, a taxa de mortalidade diminuiu para níveis semelhantes aos do período pré-pandemia da COVID-19^5^.

O protocolo SARC-HD foi publicado anteriormente e suas informações detal­hadas podem ser encontradas em outras fontes^6^. Nesta edição do JBN, Duarte et al^7^ descrevem a experiência durante as fases de recrutamento e implementação de um estudo de coorte, o SARC-HD, realizado em 19 centros de HD no Brasil. Após a conclusão das fases de recrutamento (centros e pacientes) e implementação, um questionário estruturado, tanto qualitativo quanto quantitativo, foi distribuído para coletar feedback dos principais pes­quisadores. De um total de 1.525 pacientes em HD triados, 1.008 foram incluídos no estudo multicêntrico SARC-HD, com o objetivo de explorar a sarcopenia em centros de HD em todo o país.

O objetivo deste relato inicial é descrever a experiência com o recruta­mento de centros e pacientes, bem como com a implementação do estudo SARC-HD. De acordo com os autores, a mai­oria dos centros convidados conseguiu participar e concluir a fase de implementação. A percepção deles foi de que o desenvolvimento de tutoriais e materiais padronizados foram facilitadores úteis, enquanto alguns centros descreveram a 7p-SGA como difícil de aplicar, especialmente quando não realizada por um nutricionista experiente.

A coleta sistemática de dados de pacientes submetidos à terapia renal substitutiva é fundamental para a compreensão epidemiológica e clínica do tratamento. Esses dados podem permitir um uso mais racional dos recursos econômicos e identificar intervenções para melhorar o tratamento e reduzir a morbidade e a mortalidade desses pacientes.

O Estudo Multicêntrico Brasileiro de Diálise Peritoneal (BRAZPD), um estudo de coorte observacional sobre diálise peritoneal (DP) no Brasil que coletou mensalmente dados demográficos, clínicos, laboratoriais e de desfechos de pacientes em DP tratados em 114 clínicas de diálise no país, foi iniciado em dezembro de 2004 e concluído em setembro de 2011. O BRAZPD gerou informações sólidas e importantes que têm sido bastante úteis como uma verificação da realidade da terapia de DP para todos os centros brasileiros de DP.

O BRAZPD tem incentivado jovens nefrologistas e profissionais de saúde a trabalhar com os dados, o que resultou em diversos artigos (alguns dos quais são citados aqui)^8,9,10,11^, além de dissertações de mestrado e teses de doutorado em diferentes universidades e estados do país.

Reunir profissionais de saúde de diferentes áreas do Brasil para participar de ensaios clínicos mais significativos não é um sonho. Está na hora de desenhar e realizar estudos multicêntricos que possam melhorar diretamente o atendimento aos pacientes que participam dos ensaios, bem como a todos os pacientes tratados nos mesmos centros.

Concordo plenamente com os autores quando afirmam que “apesar dos recursos limitados de pesquisa e financiamento em países como o Brasil, pesquisas multicêntricas podem ser viáveis se forem adequadamente desenhadas”.

De acordo com os autores, o principal desafio a ser enfrentado é a falta de informações padronizadas nos prontuários eletrônicos. Eu lhes faço uma pergunta: “Já pensaram em unir forças com outros grupos que realizaram ou estão realizando estudos clínicos na área de terapias renais substitutivas e compartilhar ferramentas e experiências?”

A experiência do estudo SARC-HD fornecerá informações e evidências de aplicabilidade clínica significativa. É muito interessante ressaltar que, no artigo de Duarte et al^7^ todos os investigadores principais consideraram a *publicação de artigos científicos e o aprendizado/troca de conhecimentos* como resultados importantes de sua participação no estudo SARC-HD. A mesma atitude/posição foi expressa pela maioria dos pesquisadores no estudo BRAZPD.

Enquanto isso, o estudo SARC-HD certamente está contribuindo para a compreensão da sarcopenia em nível nacional e, não menos importante, levanta questões específicas sobre sarcopenia que podem levar ao desenvolvimento de estudos cooperativos entre os diferentes registros nacionais e regionais no Brasil e no mundo.

Parabéns aos autores!
